# Trail erosion assessment and monitoring in natural areas: a comparison of traditional and high-resolution topographic surveying methods

**DOI:** 10.1007/s10661-026-15212-5

**Published:** 2026-04-09

**Authors:** Marcos Vinícius Ribeiro de Castro Simão, Estela Inés Farías-Torbidoni, Víctor Dorado, Manel Llena

**Affiliations:** 1 University of Lleida, Lleida, Spain; 2 National Institute of Physical Education of Catalonia (INEFC), Lleida, Spain; 3 Federal Institute of Education, Science and Technology of Amazonas - Campus Tefé - IFAM, Tefé, Brazil; 4 Social and Educational Research Group On Physical Activity and Sport (GISEAFE), Barcelona, Spain; 5 Fluvial Dynamics Research Group (RIUS), University of Lleida (UdL), Lleida, Spain

**Keywords:** Trail erosion monitoring, High-resolution topography, Erosion measurement methods, Recreational trail management, Forest trail management, Uncertainty assessment

## Abstract

Recreational trails in natural areas are subject to erosion, particularly when poorly planned and under visitation pressure. Accurate erosion monitoring is essential for sustainable trail management, especially in quantitative analysis requiring objective parameters of accuracy and precision. This study compares the feasibility of five erosion measurement methods applied to forest trails. The methodological approach included two traditional techniques: (i) cross-sectional area (CSA) and (ii) maximum depth (MaxD); and three high-resolution topographic (HRT) techniques: (iii) terrestrial laser scanning (TLS), (iv) structure from motion photogrammetry using an action camera (SfM-AC), and (v) an unmanned aerial system (SfM-UAS). Repeated field surveys enabled estimation of short-term erosion, in addition to historical erosion, which was assessed by comparing reconstructed pre-trail topography with current conditions. Notably, to our knowledge, this is the first study in trail management to apply check points (CPs) to quantify vertical uncertainty and integrate it into a DoD-based analysis, thereby improving the detection and interpretation of erosion processes. Results show that while CSA is practical and low cost, it may introduce undetected errors, as it does not allow for quantifying measurement uncertainty. In contrast, HRT methods produced georeferenced and accurate outputs to support spatial and temporal analyses and yielded consistent estimates of surface change. The methods differed in terms of time, financial costs, logistics, and required level of expertise. Among HRT approaches, SfM-AC emerged as the most feasible, balancing affordability, operational simplicity, and technical performance. These findings underscore the need to match erosion measurement methods to trail conditions and project objectives, and highlight the potential of photogrammetry for monitoring trail erosion in diverse environments.

**Supplementary Information:**

The online version contains supplementary material available at 10.1007/s10661-026-15212-5.

## Introduction

Trails are essential infrastructure in protected natural areas, supporting not only recreation and tourism but also research and management activities. They serve both as access routes to specific destinations, such as scenic viewpoints, waterfalls, and peaks, and as attractions in themselves, supporting activities like walking, running, and cycling (Marion, 2023). However, their continued use under growing visitation pressure presents conservation and management challenges (Spernbauer et al., 2023). Among these, erosion stands out as a critical and often irreversible impact, threatening both ecological integrity and visitor experience (Cole, 1983; Marion, 2023).

The sustainable management of trails in protected natural areas is fundamental to ensuring that these spaces continue to fulfill their social function, by promoting biodiversity conservation while simultaneously supporting a broad range of ecosystem services (Tomczyk et al., 2016). When appropriately managed, trails provide low-impact access to these environments and enhance opportunities for recreation and ecotourism, thereby promoting physical and mental well-being, environmental awareness, and appreciation for conservation (Coventry et al., 2021; Gobster et al., 2023; Marion, 2023). However, growing visitation, combined with poor trail planning and lack of maintenance, has raised concerns about natural resource impacts and the decline in the quality of the visitor experience (Farías-Torbidoni et al., 2023; Spernbauer et al., 2023).

Trails inevitably undergo morphological changes due to user-related pressures, environmental characteristics, and managerial factors (Olive & Marion, 2009). In addition, recreational use of trails frequently leads to negative impacts on surrounding trail-related ecosystems (Simão et al., 2025). Human presence may disturb wildlife behavior (Miller et al., 2020), reduce vegetation cover through trampling (Thurston & Reader, 2001) or wood collection in campsites (Marion, 2016), and contribute to the spread of invasive species and the contamination with human waste (Marion & Leung, 2011; Pickering et al., 2010). Some of these impacts can be mitigated through management practices, education, or access restrictions (Hockett et al., 2017). Nonetheless, trail erosion, changes in drainage patterns, and sediment loss are frequently considered irreversible or prohibitively expensive to restore (Bratton et al., 1979; Ng et al., 2017; Olive & Marion, 2009; Salesa & Cerdà, 2020; Tomczyk et al., 2023), given that soil formation is a process of long timescales (Salesa & Cerdà, 2019). Beyond ecological damage, eroded trails also compromise safety, scenic quality, and the overall visitor experience (Cole, 1983; Marion, 2023).

Since the 1970s, scientific literature has reported that recreational use of trails leads to these impacts, prompting the development of techniques to monitor these disturbances (Coleman, 1977; Jewell & Hammitt, 2000; Marion, 2016). For decades, erosion measurement has primarily relied on the cross-sectional area (CSA) method, a simple and cost-effective approach (Coleman, 1977; Leonard & Whitney, 1977; Weaver & Dale, 1978). This method, described extensively in the literature, involves measuring the trail’s cross-sectional profile at multiple locations and can be used to estimate both historical and recent erosion (Cole, 1983; Coleman, 1977). Historical erosion is assessed by comparing the current trail tread with the presumed original topography, typically approximated by interpolating between the highest points at the trail’s edges. In contrast, recent erosion is calculated as the change in trail tread topography between successive measurements taken over a known time interval.

Simplified adaptations of the CSA method have been proposed to minimize fieldwork. Olive and Marion (2009) limited measurements to depths surpassing a predefined threshold, though this approach was found to underestimate erosion. Rinehart et al. (1978) also proposed the use of CSA as a metric for assessing trail erosion. Instead of relying on manual field measurements with rulers, they applied stereo photography to estimate cross-sectional area, yielding results comparable to those obtained through direct measurement. Sampling design in CSA-based studies varies considerably, being often systematic, random, or purposive, depending on management priorities (Cole, 1983). Spacing intervals between cross-section measurements also differ among studies. While some authors recommend a distance of 100 m as optimal (Leung & Marion, 1999), trail morphological factors may influence the appropriate interval between measurements. Based on these samples, average CSA values are extrapolated along the entire trail length to estimate total soil loss, typically expressed as volume per unit area or linear extension (e.g., Smith & Pickering, 2025; Tomczyk & Ewertowski, 2013), or as mass per unit area (e.g., Salesa & Cerdà, 2019).

The CSA method is well established in the trail management literature and is useful for estimating overall trail condition (Cole, 1983; Salesa & Cerdà, 2020). It is often argued that erosion too minor to be detected with this method is also too negligible to pose a practical concern (Coleman, 1977). However, the CSA method has known limitations in detecting subtle changes. One key limitation is that changes can only be identified directly beneath each transect, making it difficult to detect features such as continuous rill lines or elongated erosion channels, as well as patterns of sediment accumulation along the trail axis. Furthermore, because the CSA method uses measurements at 10-cm intervals, it is a discrete approach that may miss small-scale variations between points. Another limitation is the method’s susceptibility to human error during manual measurements, especially when baseline and follow-up surveys are performed by different individuals (Cole, 1983). Despite these limitations, the CSA method remains widely used for identifying management-relevant problem areas due to its low technical expertise required. However, when the goal is to detect subtle, millimetric morphological changes or generate detailed three-dimensional representations, as often necessary in scientific studies of erosion processes and patterns, more precise methods may be required. Trail management could greatly benefit from emerging measurement techniques that offer enhanced accuracy, precision, and operational efficiency (Marion & Leung, 2001; Tomczyk et al., 2023).

In this context, the growing availability and application of remote sensing (RS) techniques enabling the generation of high-resolution topography (HRT) have prompted researchers to explore their potential for measuring and monitoring soil erosion on trails (e.g., Llena, 2025; Tomczyk, 2011; Tomczyk et al., 2023). Until recently, studies investigating erosion and deposition on trails were based exclusively on cross-sectional-based measurements. The study by Tomczyk and Ewertowski (2013), which quantified short-term surface changes on recreational trails using topographic surveys and digital elevation models of difference (DoD), was the first to adopt an area-based approach. This advancement enabled the extraction of as many cross-sections as needed and improved the spatial and temporal analysis of topographic change distribution along the trail, enhancing the detail and flexibility of erosion assessments.

In the same line, Salesa et al. (2020) were the first to apply structure from motion (SfM) photogrammetry to erosion research on recreational trails, comparing estimates from traditional methods with those obtained using this HRT technique. They assessed the CSA method alongside a simplified method based solely on the deepest erosion point. In addition, they evaluated HRT approaches using SfM techniques with imagery captured by an unmanned aerial system (UAS) and a smartphone. More recently, Hayakawa et al. (2025) applied terrestrial SfM combined with light detection and ranging (LiDAR) on small-scale transects (6 m) in open areas without canopy cover to measure the effects of ground-contacting gear on the trail surface with centimeter-level accuracy. Other studies employing airborne LiDAR to assess erosion at broader scales have also pointed to ground-based LiDAR as a promising tool for monitoring trail conditions over time (Eagleston & Marion, 2020). Yet, uncertainties remain regarding the broader applicability of such advanced methods in practical trail monitoring contexts. Despite the growing availability of HRT methods for erosion assessment, few studies have systematically compared their accuracy, precision, and feasibility. Factors such as data collection time, processing demands, costs, and bureaucratic constraints are often overlooked, limiting the applicability of these techniques in both scientific research and real-world trail management.

To address this gap, this study aimed to evaluate the feasibility of different erosion measurement methods in forested trail environments under recreational use. We hypothesized that HRT techniques would yield more precise and accurate erosion estimates than traditional methods, but with varying levels of feasibility, and that they could additionally produce erosion and deposition maps to support spatial analysis of trail surface change. To test these hypotheses, we applied five erosion measurement methods to two trail segments, collecting data at two time points spaced 34 days apart. Additionally, historical erosion was assessed by comparing reconstructed pre-trail topographic conditions with the current situation.

## Materials and methods

### Study area

**Fig. 1:**
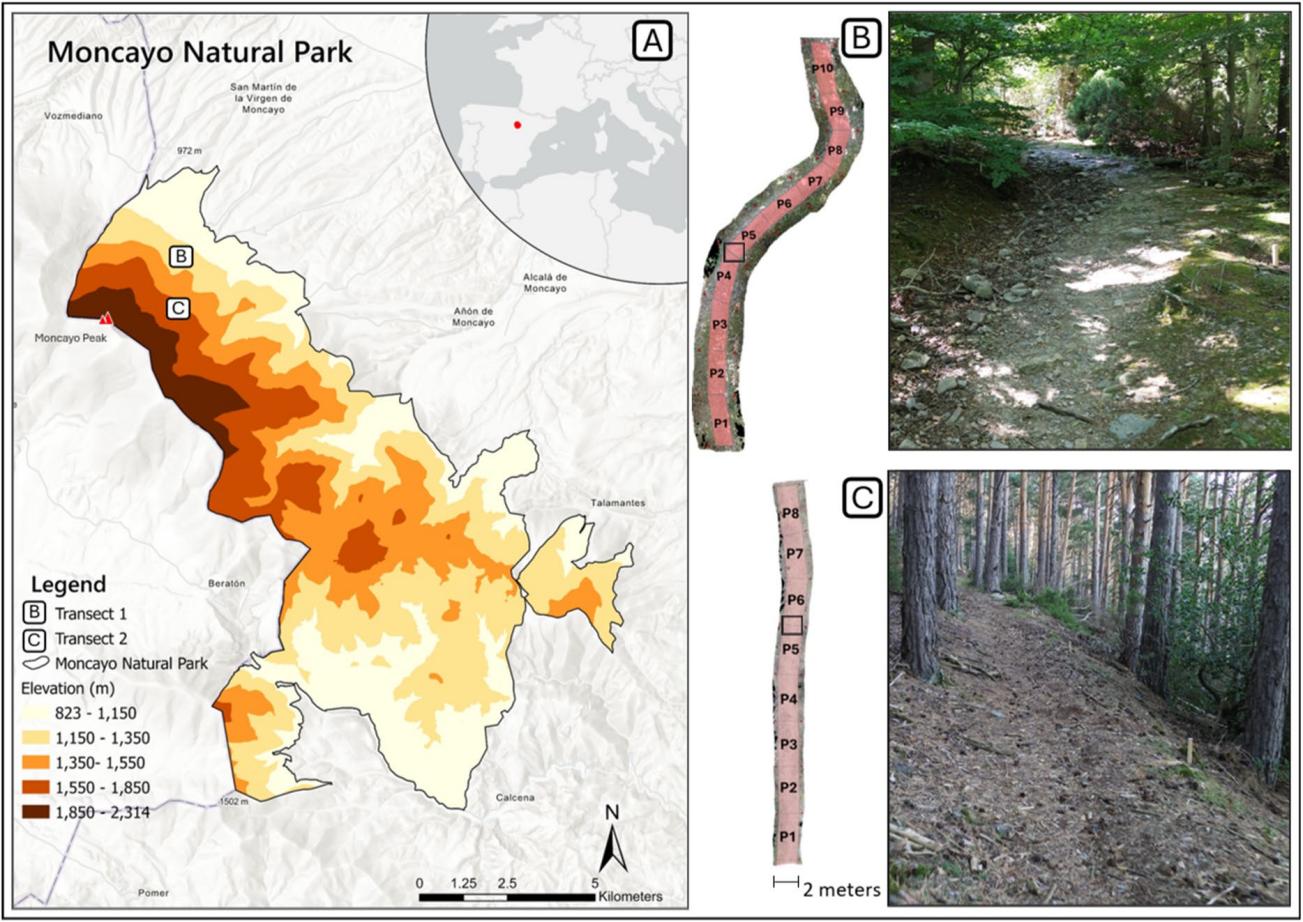
Study area. **A** Location of Moncayo Natural Park, with inset showing its position within Spain and Europe. **B** Sampling plots over the orthomosaic of Transect 1 (left), with black rectangle indicating the area shown in the photo (right), featuring exposed roots and rocks from millimetric fragments to large boulders. **C** Sampling plots over the orthomosaic of Transect 2 (left), with black rectangle indicating the photo area (right), showing fewer exposed elements but abundant pine needles

The data were collected from two trails located in the Moncayo Natural Park (112 km^2^), situated on the north-western part of the Iberian System, in Spain, with elevations ranging from 823 to 2314 m above sea level (a.s.l.) at the Moncayo Peak (Fig. 1). The two trails differed in terms of surrounding vegetation types and trail surface characteristics, providing contrasting conditions for erosion assessment. The annual mean precipitation can reach 1500 mm at high elevations and can drop below 400 mm at lower elevations (Martínez del Castillo et al., 2019). During the monitoring period, the study area experienced heavy precipitation totaling 116.6 mm. Notably, two intense rainfall events exceeding 25 mm∙day^−1^ occurred during the monitoring period, on September 20, 2025 (25.2 mm) and October 2, 2025 (33.6 mm).

Located within a climatic transition zone between the Mediterranean and Eurosiberian biogeographical regions, the park features a diverse range of vegetation types. Along its altitudinal gradient, forest formations are primarily composed of Pyrenean oak (*Quercus pyrenaica* Willd.) at lower elevations, and Scots pine (*Pinus sylvestris* L.) and European beech (*Fagus sylvatica* L.) at mid to higher elevations (Martínez del Castillo et al., 2019). The park features a network of primary and secondary access roads, paths, and trails. Trail use is restricted to pedestrians, while bicycles, horses, and motorized vehicles are not permitted on trails (Government of Aragón, 2014).

### Transect allocation and plot distribution

Transect 1 is located along a trail in a deciduous forest dominated by European beech (*Fagus sylvatica* L.), featuring a trail tread approximately 3.5 m wide with abundant exposed roots and rocks. Transect 2 is situated along a trail in a pine forest (*Pinus* spp.) with a narrower tread (2.5 m), fewer exposed roots and rocks, and a prominent layer of pine needles deposited along the trail surface (Martínez del Castillo et al., 2019).

Each transect was defined as a 2-m-wide strip along the central portion of the trail, based on the space most affected by hiker traffic and surface runoff. Transect 1 measured 50 m in length (1206–1215 m a.s.l., avg. slope: 18%), and Transect 2 measured 40 m (1562–1570 m a.s.l., avg. slope: 22%) (Table 1). Trail edges were determined based on signs of long-term trampling, such as subtle microtopographic changes. These boundaries were then used to guide the placement of wooden stakes marking the transects.

**Table 1:** Characteristics of the sampled trails and the respective transect allocated in each trail

Transect	Trail width (m)	Trail slope	Transect width (m)		Transect length (m)	Transect area (m^2^)	Number of plots	Main caracteristics
1	~3.5	18%		2	50	103.2	10	Deciduous forest dominated by European beech, with a trail tread characterized by abundant fixed stones, loose rocks, and exposed roots
2	~2.5	22%		2	40	78.87	8	Coniferous forest dominated by Scots pine; trail tread with few loose stones and accumulation of pine needles

### Data collection and processing

Eleven pairs of stakes were installed along Transect 1 and nine along Transect 2, positioned approximately every 5 m at the trail edges. Each stake (50 cm long, 3 cm diameter) was embedded 30 cm into the soil, with 20 cm remaining visible. These served both as reference points for CSA and MaxD measurements and as ground control points (GCPs) for the HRT methods.

The transects were delineated in ArcGIS Pro by connecting central points between each pair of stakes to form a midline, which was then buffered by 1 m on each side to define the 2-m-wide study area. Each transect was subdivided into plots (10 in Transect 1 and 8 in Transect 2), each approximately 5 m in length. Plot boundaries corresponded to the cross-sectional measurement lines between consecutive stake pairs. All erosion and deposition values were standardized per square meter to allow consistent comparisons across the plots.

The transects were sampled on September 17 (hereafter referred to as “initial”) and October 21, 2024 (hereafter referred to as “latest”), resulting in a 34-day monitoring period. During this period, the park remained open to visitors, and hikers were observed on the trails; however, it was not possible to record the number of trail users due to a malfunction in the automatic counting equipment. Nevertheless, substantial changes in the trail tread were observed in the field along both trails, primarily due to the two intense rainfall events that occurred during this interval.

**Fig. 2:**
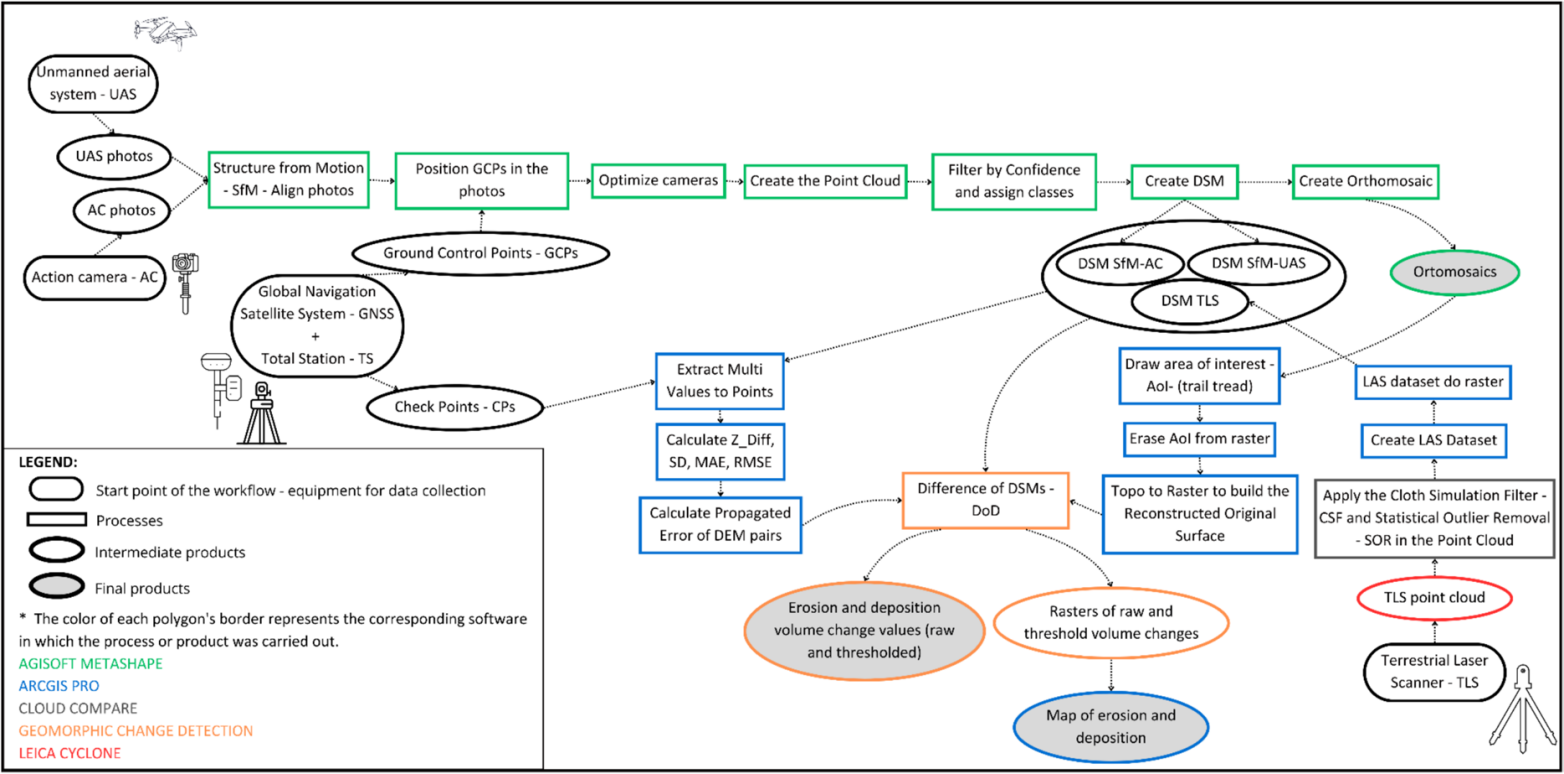
Workflow diagram outlining the step-by-step procedures for each HRT method, from field data collection to data processing. The legend indicates the starting points of each workflow, processes, intermediate products, and final products (i.e., the results presented in this article). The colors outlining each polygon represent the software used for each corresponding process or product

Five methods were used to measure erosion in this study: cross-sectional area (CSA), maximum depth (MaxD) of the CSA, structure from motion with action camera photos (SfM-AC), structure from motion with UAS camera photos (SfM-UAS), and terrestrial laser scanning (TLS). From the five methods, only CSA, MaxD, and SfM-UAS were used for the initial measurement, whereas all five methods were applied in the latest measurement. The data collection procedures for each method are detailed in the following sections. Figure 2 presents a step-by-step flowchart detailing the data collection and processing procedures for the HRT methods.

### Cross-sectional area and maximum depth

**Fig. 3:**
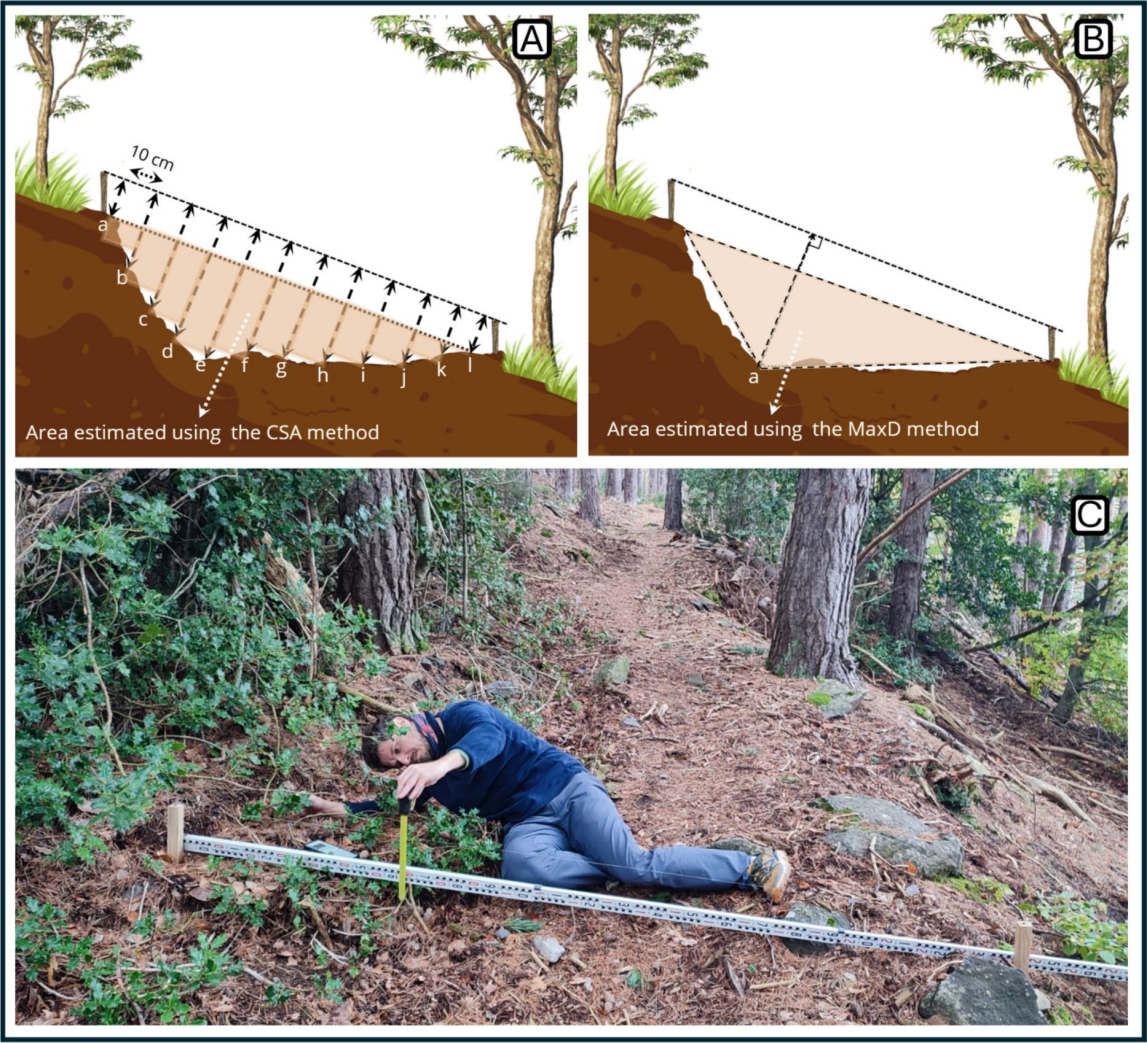
**A** Example of measurement using the cross-sectional area (CSA) method. The height of the stakes was first recorded, followed by vertical distance measurements taken every 10 cm perpendicular from the nylon line to the trail surface at points “a,” “b,” and so on, until reaching the opposite stake. **B** Example of measurement using the maximum depth (MaxD) method. The height of the stakes was first recorded, followed by a single vertical measurement taken from the nylon line to the trail surface at the point of maximum depth “a”. **C** Photograph of the measurement being carried out using the CSA method

For the measurement of CSA and MaxD, a nylon line was tied between the ends of each pair of stakes. A nylon line was used instead of a metal bar, as the weight of the metal often causes it to sag in the middle, which could result in underestimating depth readings. Parallel to the nylon line, an aluminum rod graduated in millimeters was placed as a reference for vertical measurements, which were taken every 10 cm, starting at one stake and ending at the opposite stake across the trail (Coleman, 1977; Marion & Wimpey, 2017). Measurements were taken using a steel tape measure, also graduated in millimeters, stretched perpendicularly (at a 90° angle) to the line until it touched the ground surface directly beneath it (Fig. 3).

From each vertical measurement taken, the height of the stakes was subtracted to consider only the area from the ground level at the trail edges, corresponding to the actual eroded area. These measurements were referred to as *depths*. When the stakes in each pair had different heights, a correction factor was applied to the field measurements to account for the slope of the line. The cross-sectional area between each pair of stakes was then calculated using the following formula:

$$Cross\;sectional\;area\;(CSA)=\sum\limits_{i=0}^n\left(\frac{D_i+D_{i+1}}2\right)\times0.0001$$

where *D*_*i*_ is the depth measured at point *i*, *D*_*i*+1_ is the depth measured 10 cm after point *i*, and 0.0001 is the conversion factor from square centimeters to square meters.

### Structure from motion with action camera and UAS photos

**Fig. 4:**
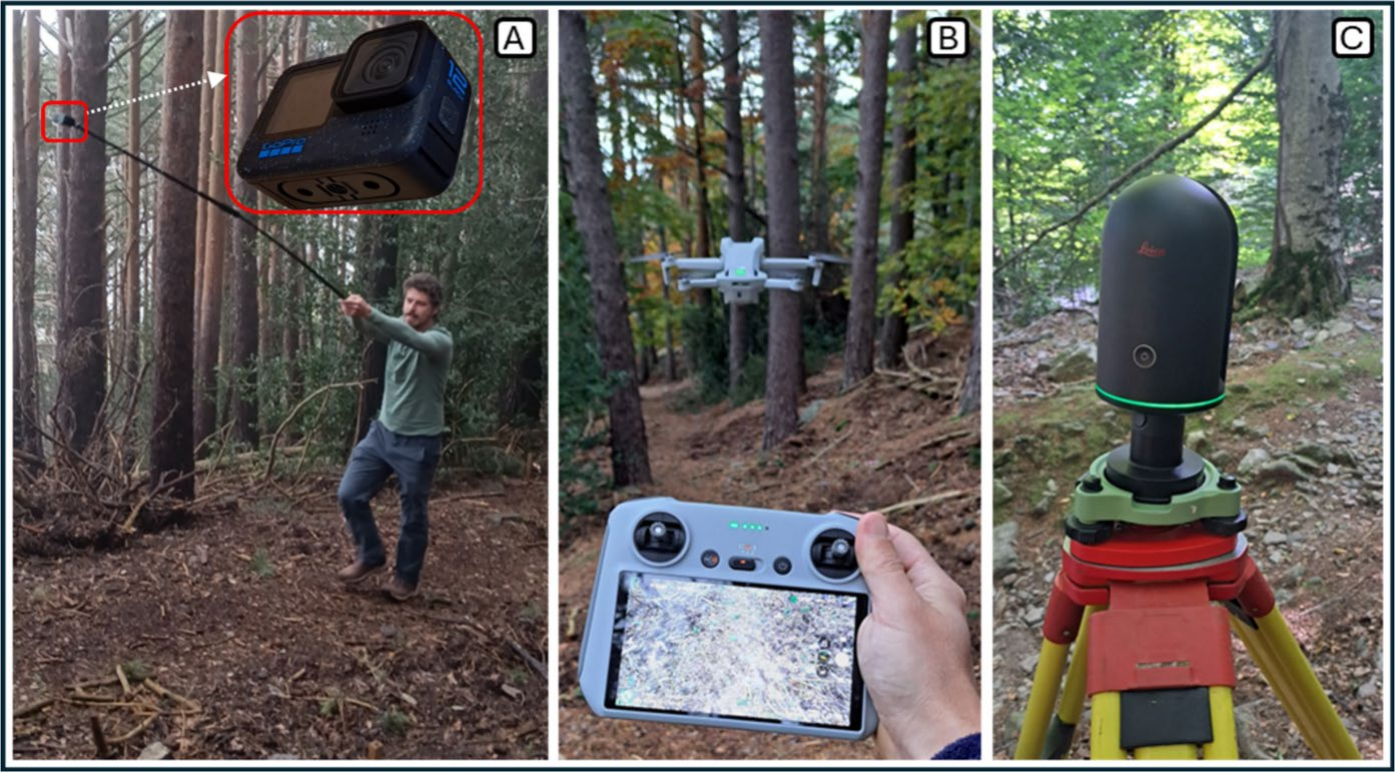
**A** Image acquisition using the action camera mounted on the pole (inset shows a close-up of the GoPro camera). **B** Image acquisition using the UAS. **C** Point cloud acquisition using the TLS

Structure from motion (SfM) was performed using two different image acquisition platforms: an action camera and a UAS (Fig. 4A, B). In the first case, an action camera GoPro Hero 12 Black with 35-mm equivalent focal length, 156° field of view, and f/2.5 (see Table 1 in Supplementary data for details) was mounted on a 1.5-m pole and programmed to automatically capture photos every 2 s in linear mode. The operator slowly walked along the transect capturing images in both directions, over six routes. On average, 95 photos were taken per pass, each photo covering an area of approximately 16 m^2^.

In the second case, a UAS DJI Mini 3 Pro with 24 mm equivalent focal length, 82.1° field of view, f/1.7, and focus range from 1 m to ∞ (see Table 1 in Supplementary data for details) was used. The camera was set to capture photos every 2 s (interval timer, auto shooting). The UAS completed six routes along the transect, capturing photos during outbound and return flights. It maintained an average altitude of approximately 2.5 m, with variations between 1.5 and 4 m due to obstacles such as tree branches and shrubs obstructing the trail corridor. On average, 92 photos were taken during each pass. For both the action camera and UAS setups, a minimum overlap of 70% between sequential images was ensured (Carrivick et al., 2016), with average overlaps of approximately 87% and 82%, respectively.

The photos were processed in Agisoft Metashape Professional, version 2.1.2 build 18,204 (64-bit), where they were aligned. The projected coordinates of the ground control points (GCPs), referenced to ETRS89 - UTM zone 30 N (EPSG: 25830), were then imported and positioned. The root mean square error (RMSE) of the GCPs was 0.012 m for both platforms at Transect 1 (T1_UAS and T1_AC), and 0.008 m for both platforms at Transect 2 (T2_UAS and T2_AC). Then, the Optimize Camera Alignment function was applied to perform a full bundle adjustment, refining both the exterior (camera position and orientation) and interior (camera calibration) parameters, as well as the tie point coordinates. The following interior parameters were selected for optimization: Fit f, Fit cx, cy, Fit k1, Fit k2, Fit k3, Fit p1, and Fit p2 (Agisoft, 2025).

Next, a dense point cloud was generated using depth maps as source data, with high quality and aggressive depth filtering. The resulting point cloud had an average density of 55 points∙cm^−2^, with 53 points∙cm^−2^ for the action camera and 56 points∙cm^−2^ for the UAS. To remove unreliable or noisy points (e.g., vegetation or artifacts), a confidence-based filter was applied, with a minimum threshold of 3 and a maximum of 255. Although no formal ground classification was performed, the remaining points after filtering were considered to represent ground surfaces for the purpose of generating the digital surface model (DSM). Based on the filtered point cloud, a filtered DSM was generated with a 1-cm raster resolution. An orthomosaic was also produced with an average pixel size of approximately 1 mm. Data processing was performed on a laptop running Windows 11 64-bit, equipped with 64 GB of RAM, an Intel(R) Core(TM) Ultra 9 185H CPU, and an NVIDIA GeForce RTX 4070 GPU.

### Terrestrial laser scanner

A TLS (Leica BLK360) with a capture speed of 680,000 points per second, and a 3D accuracy of 4 mm at 10 m and 7 mm at 20 m, was used (Leica Geosystems, 2025a). It was first positioned at the beginning of each transect and then approximately every 8 m along its length, resulting in six data collection points (Fig. 4C). To capture a full 360° point cloud and to ensure that the blind spot formed beneath the scanner did not overlap with the trail tread, the device was placed at the trail margins.

The point clouds obtained with the TLS were processed using the software Leica BLK Data Manager, in which multiple datasets were registered using the GCPs network, and subsequently exported in LAS format. This point cloud was then imported into CloudCompare version 2.13.2 (CloudCompare, 2024), where the Cloth Simulation Filter (CSF) (Zhang et al., 2016) and the Statistical Outlier Removal (SOR) filter were used to remove points not corresponding to the ground surface. Finally, a DSM was obtained from the filtered point cloud with a pixel size of 1 cm.

### Ground control points network

A fixed network of ground control points (GCPs) was established using stakes permanently installed along the edges of each transect, serving as stable references throughout the study period. Their positions were first surveyed using a total station (TS) (Leica TPS1201+ TPS), which provided millimetric-scale positional accuracy (i.e., 0.0017 m of average precision). This high precision significantly reduces positioning errors typically associated with floating GCP networks, thereby lowering the minimum level of detection (minLoD), an essential requirement when working with fine-scale erosion and deposition estimates (e.g., Llena et al., 2020).

To georeference this control network, a subset of reference points located approximately 30 m from the trail was surveyed using a Leica Viva GS15 GNSS-rtk (Leica Geosystems, 2025b). These reference points were then used to transform the locally surveyed stake network into a globally projected coordinate system ETRS89/UTM zone 30 N (EPSG: 25830), using the EGM08REDNAP geoid model. These georeferenced stakes were used as ground control points (GCPs) for both the SfM and TLS data.

### Check points (CPs) network

A set of 100 check points (CPs) was surveyed along each transect tread using a total station, resulting in a control dataset used to evaluate the accuracy of the digital surface models (DSMs). The DSMs generated by the three HRT methods were imported into ArcGIS Pro (version 3.3.2, ESRI, 2025). The error, defined as the elevation difference (hereafter referred to as Z diff) between the observed values (TS) and the predicted values (DSM), was calculated as follows:

$${Z\;diff}_i=({Z\;value\;TS}_i-{Z\;value\;DSM}_i)$$

in which *Z value TSi* refers to the altitude value of check point *i* obtained with the total station (TS), and *Z value DSMi* refers to the modeled altitude value corresponding to check point *i*.

Following Smith and Vericat (2015), the *Z* diff values were employed to evaluate the accuracy via mean absolute error (MAE) and root mean square error (RMSE), and precision via standard deviation (SD) of the HRT methods. It is important to note that *Z* diff values were computed only for the latest field survey, using the DSMs generated during this latest measurement.

### Comparison of the feasibility of topographic surveying methods

The comparison of topographic surveying methods was conducted in two complementary stages. First, the consistency of erosion estimates produced by each method was evaluated across two temporal scales: long term (historical) and short term (monthly). Second, the overall feasibility of each method was assessed using both quantitative and qualitative criteria.

### Estimation of erosion using traditional methods

For the CSA method, historical erosion volume for each plot was calculated based on the latest measurements. As CSA inherently simulates the terrain’s topography prior to trail formation, it directly estimates cumulative (historical) erosion. Erosion volume was computed by multiplying the average of two consecutive CSA measurements (taken at the beginning and end of each plot) by the plot length (see Table 2 in Supplementary data for details). This value was then divided by the plot area to express erosion per square meter. The calculation followed the equation:

$$Erosion\;(CSA)\,(\mathrm{m}^3\times\mathrm{m}^{-2})=\left(\frac{{CSA}_i+{CSA}_{i+1}}2\right)\times{Length}_i\times{Area}_i^{-1}$$

where ${CSA}_{i}$ is the cross-sectional area value at position *i* (the start of the plot), in m^2^, ${CSA}_{i+1}$ is the cross-sectional area value at the subsequent position (the end of the plot), in m^2^, ${Length}_{i}$ is the length of the plot *i*, in meters, and ${Area}_{i}$ is the area of the plot *i*, in m^2^.

The MaxD method followed the same procedure, using the maximum depths recorded at the beginning and end of each plot. Assuming a triangular incision shape, the cross-sectional area was estimated by multiplying the maximum depth (triangle height) by the transect width (triangle base) and dividing by two. The erosion volume was then computed as with the CSA method. The calculation followed the equation:

$$Erosion\;(MaxD)(\mathrm{m}^3\times\mathrm{m}^{-2})=\left(\frac{{MaxD}_i+{MaxD}_{i+1}}2\right)\times Twidth\times2^{-1}\times\;Length\times{Area}^{-1}$$

where ${MaxD}_{i}$ is the maximum depth value at position *i* (the start of the plot), in meters; ${MaxD}_{i+1}$ is the maximum depth value at the subsequent position (the end of the plot), in meters; $Twidth$ is the transect width (2 m); $PLenth$ is the plot length, in meters; and $Area$ is the plot area, in square meters (m^2^).

For both CSA and MaxD methods, monthly erosion was calculated by subtracting the erosion value obtained in the initial measurement from that obtained in the latest measurement.

### Estimation of erosion using HRT methods

**Fig. 5:**
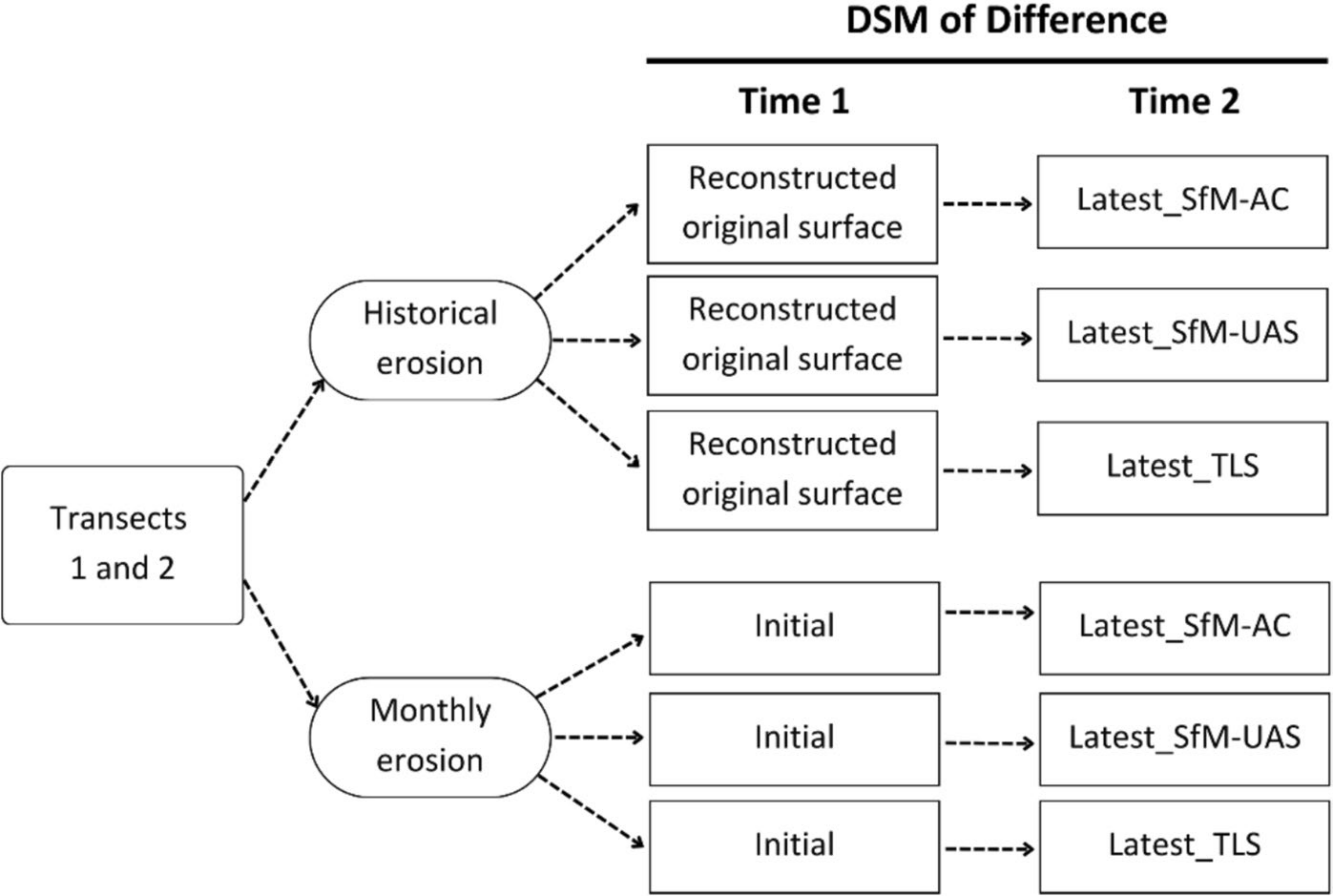
Workflow for estimating historical and monthly erosion using the DSM of Difference (DoD) approach. For historical erosion, the reference surface (Time 1) is a reconstructed original surface based on the SfM-UAS DSM. For monthly erosion, the initial surface (Time 1) is the SfM-UAS DSM from the first survey. In both cases, Time 2 corresponds to the most recent DSMs generated with SfM-AC, SfM-UAS, and TLS, acquired 34 days after the initial survey

Historical and monthly erosion estimates using HRT methods were derived via the DSM of Difference (DoD) approach (e.g., Wheaton et al., 2010). For both analyses, the baseline surface (Time 1) was generated using SfM-UAS, as it was the only method available at the initial survey. For historical erosion, the reference surface (Time 1) was the reconstructed original surface, based on the initial SfM-UAS-derived DSM, and the comparison surfaces (Time 2) were the most recent DSMs generated by each HRT method (latest SfM-AC, SfM-UAS, and TLS). For monthly erosion, Time 2 represented DSMs captured 34 days later by each method. This procedure was applied to both Transects 1 and 2 (see Fig. 5).

The reconstructed original surface DSM, representing pre-disturbance topography, was generated in ArcGIS Pro based on SfM-UAS-derived DSMs. To reconstruct the trail-free surface, the main trail tread area (i.e., the section between stake pairs) was removed using the Erase tool. The remaining DSM, containing only the trail margins, was then interpolated using the Topo to Raster tool. Any gaps in the interpolated surface were subsequently filled using the Fill tool. This process resulted in a reconstructed surface representing conditions prior to trail formation (e.g., Tomczyk et al., 2023).

For each method, the reconstructed original surface, the initial and latest DSMs, and a shapefile delineating plot boundaries within each transect (see pink polygons in Fig. 1) were imported into Geomorphic Change Detection (GCD) software (Wheaton et al., 2010). DoD analyses were then performed to estimate both historical and monthly erosion. To account for uncertainty in the elevation data, a minimum level of detection (minLoD) was applied, ensuring that only elevation changes exceeding this threshold were considered real topographic changes, as opposed to variations attributable to measurement noise or error (Anderson, 2019). The minLoD was calculated based on propagated uncertainty from the input DSMs. The minLoD values applied in each dataset are reported in Table 3 (Supplementary Data).

Since the mean absolute error (MAE) was computed only for the latest measurement, the same MAE values were applied to the initial and latest DSMs when estimating the propagated error. The propagated error was then calculated using the following formula:

$${\upsigma }_{DoD}=\sqrt{{{(\upsigma }_{DEM\_initial})}^{2}+{{(\upsigma }_{DEM\_lastest})}^{2}}$$

where ${\upsigma }_{DoD}$ is the total propagated error in the DoD, ${\upsigma }_{DEM\_initial}$ is the uncertainty (MAE) of the initial DSM, and ${\upsigma }_{DEM\_latest}$ is the uncertainty (MAE) of the latest DSM.

The DoD analysis resulted in erosion and deposition volumes, along with corresponding DSMs, for both raw data (without error filtering) and net data (thresholded by propagated error). Throughout this article, all reported erosion and deposition values refer to net results. Orthomosaics were generated by projecting the georeferenced images onto the SfM-derived DSMs. These orthomosaics were then overlaid with the DoD raster maps (erosion/deposition) to support visual interpretation of microtopographic changes. This overlay helped identify spatial patterns of erosion and deposition along the trails, particularly where changes were subtle or localized.

### Overall feasibility

The feasibility of each method was evaluated through a combination of quantitative and qualitative criteria. Quantitative parameters were tailored to the capabilities of each method and included consistency of erosion estimates across two temporal scales—long term (historical) and short term (monthly). For the HRT methods, the evaluation considered fieldwork and data processing time, equipment cost, and operational constraints. Additional metrics included vertical accuracy and precision, sensitivity to topographic change (i.e., minimum level of detection), ground and orthomosaic resolution (mm∙pixel^−1^), DSM resolution (mm∙pixel^−1^), and point density (points∙cm^−2^). In contrast, the traditional methods do not enable the extraction of these additional metrics, so the analysis was limited to fieldwork duration, data processing time, and equipment cost. Qualitative criteria included the need for trained personnel, bureaucratic restrictions, and logistical or operational challenges encountered during implementation. These factors, combined with the quantitative metrics, supported a comprehensive evaluation of each method’s feasibility, strengths, and limitations for trail monitoring applications.

### Statistical analysis

All statistical analyses were conducted using R (R Core Team, 2024). The dataset contained erosion measurements obtained from five methods across 10 plots in Transect 1 and 8 plots in Transect 2. Because the same plots were measured two times using different methods, the data exhibit a hierarchical structure, where observations within the same plot are likely more similar to each other than to observations from different plots. To account for this structure, a linear mixed model (LMM) was fitted using the lme4 package (Bates et al., 2015), with erosion as the response variable and method as a fixed effect. The plot identifier (plot) was modeled as a random effect, capturing variability across plots. The model was specified as follows:

$${Y}_{ij} = {\beta }_{0}+ {\beta }_{1}{X}_{ij}+\left(1{Plot}_{j}\right)+ {\varepsilon }_{ij}$$

where ${Y}_{ij}$ is the erosion measurement for observation *i* in plot *j*, ${X}_{ij}$ represents the method used, ${\beta }_{0}$ and ${\beta }_{1}$ are fixed-effect parameters, ($1{Plot}_{j}$) is the random intercept, and ${\varepsilon }_{ij}$ is the residual error, assumed to be normally distributed.

After fitting the model, the assumptions of normality and homoscedasticity were assessed, as these are key assumptions underlying the use of ANOVA for hypothesis testing. Residual normality was evaluated using the Shapiro-Wilk test and by inspecting a Q-Q plot; homoscedasticity was checked using Levene’s test and by examining a residual plot for any systematic deviations. A Type III ANOVA was performed to test whether method had a significant effect on erosion. Although the dataset was balanced, the Satterthwaite approximation was used to estimate denominator degrees of freedom, as it accounts for the hierarchical structure of the mixed-effects model. The likelihood ratio test (ANOVA) was used to assess the significance of the fixed effect.

To determine which measurement methods differed significantly, Tukey’s Honest Significant Difference (HSD) test was applied using the emmeans package (Lenth, 2025). Pairwise comparisons were conducted through estimated marginal means (emmeans()), adjusting for the random effect of plot to ensure accurate estimations of method differences. Statistical significance was set at *α* = 0.05.

## Results

### Assumption testing and model validation

The Shapiro-Wilk test results indicated normality regarding historical erosion in Transect 1 (*p*-value = 0.1281) and monthly erosion in Transect 2 (*p*-value = 0.1178). However, they indicated a lack of normality for monthly erosion in Transect 1 (*p*-value = 0.02674) and historic erosion in Transect 2 (*p*-value = 0.01587), although the Q-Q plot did not reveal significant deviations from normality, suggesting that the data’s distribution is not severely non-normal. Similarly, Levene’s test indicated homoscedasticity (*p* > 0.05) for historical erosion in Transect 1 (*p*-value = 0.1915) and monthly erosion in Transect 2 (*p*-value = 0.06971), but showed some heteroscedasticity (*p*-value < 0.05) for monthly erosion in Transect 1 (*p*-value = 0.004903) and for historic erosion in Transect 2 (*p*-value = 4.636e^−05^) (see Table 4 in Supplementary data). Therefore, we decided to proceed with further analyses. Consequently, we conducted Welch’s ANOVA and pairwise *t*-tests without assuming equal variances in R, specifically targeting monthly erosion in Transect 1 and historical erosion in Transect 2. The consistency between these additional test results and those obtained from Tukey’s HSD test (see Table 5 in Supplementary data) corroborated our belief in the reliability of this former test. Thus, despite the non-normality of residuals and the heteroscedasticity regarding monthly erosion in Transect 1 and historic erosion in Transect 2, the alignment of outcomes from the subsequent analyses led us to rely on the ANOVA and Tukey’s HSD test results for our final evaluations and discussions.

### Historical and monthly erosion volume estimates

The statistical analyses revealed significant variations in erosion estimates (see Table 5 in Supplementary data for more details) depending on the surveying method and the transect analyzed. The choice of method had a significant effect on historical erosion estimates in Transect 1 (*F*(4, 36) = 65.58, *p* < 0.001), indicating substantial differences across methods. The choice of method also had a significant effect on monthly erosion estimates in Transect 1 (*F*(4, 36) = 7.59, *p* < 0.001), although the lower *F*-value compared to historical erosion indicates less pronounced variation between methods.

For historical erosion in Transect 2, the effect of surveying method was also significant (*F*(4, 28) = 2.99, *p* = 0.036), although the lower *F*-value suggests that method-dependent differences were less pronounced than in Transect 1. In contrast, no significant differences were found for monthly erosion in Transect 2 (*F*(4, 28) = 1.06, *p* = 0.39), suggesting that, for this transect, monthly erosion estimates were consistent across measurement approaches.

**Fig. 6:**
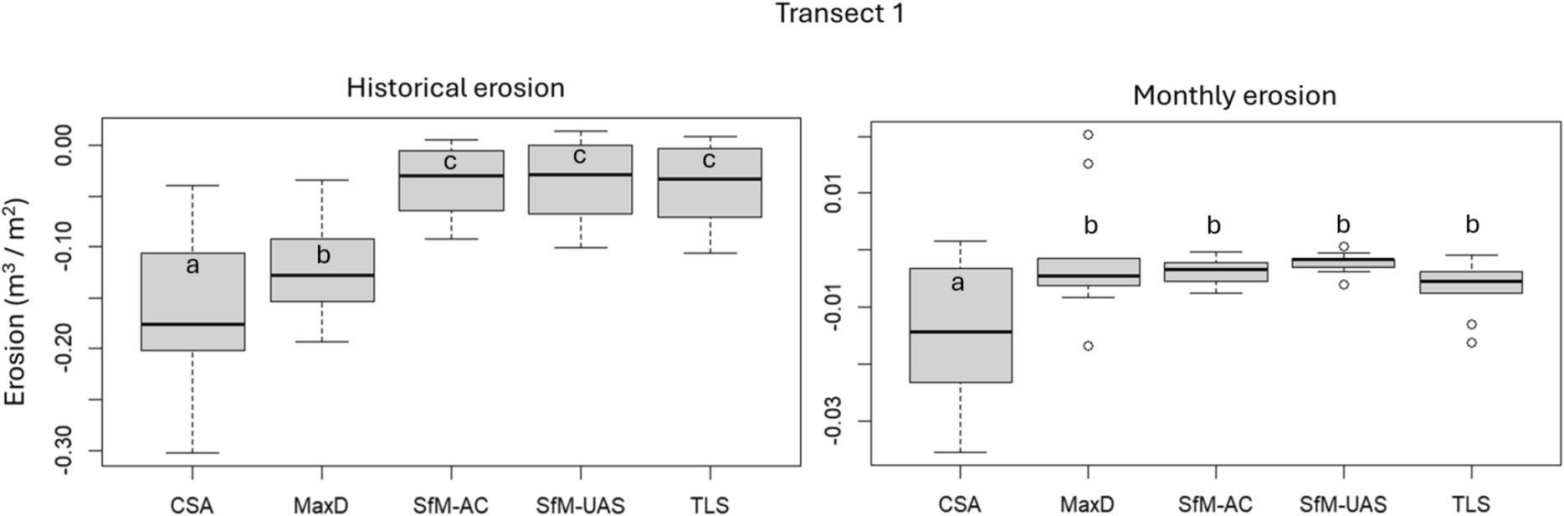
Boxplots showing erosion rates (m^3^∙m^−2^) estimated by different methods in the Transect 1, for historical and monthly erosion. Letters indicate statistical significance based on Tukey’s HSD test: methods sharing the same letter are not significantly different, whereas different letters indicate significant differences (*p* < 0.05)

Tukey’s test revealed that most pairwise comparisons for historical erosion in Transect 1 were significantly different (*p* < 0.001), except among HRT methodologies (SfM-AC vs. SfM-UAS, SfM-AC vs. TLS, SfM-UAS vs. TLS) which did not differ significantly. Similarly, for monthly erosion in Transect 1, significant differences were observed between CSA and all other methods (*p* < 0.05), while comparisons among MaxD and the HRT methods were not statistically significant (Fig. 6).

**Fig. 7:**
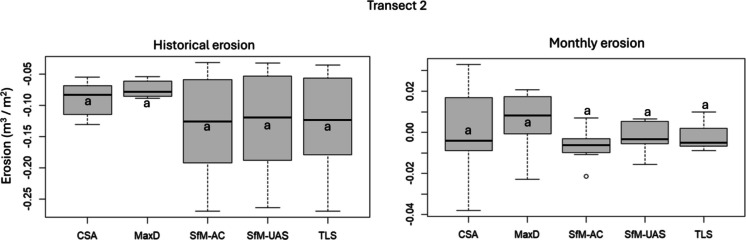
Boxplots showing erosion rates (m^3^∙m^−2^) across different surveying methods in Transect 2. Letters indicate statistical significance based on Tukey’s HSD test: methods sharing the same letter are not significantly different

In contrast, although ANOVA indicated a significant overall effect for historical erosion in Transect 2, none of the pairwise comparisons showed statistically significant differences. Similarly, for monthly erosion in Transect 2, all pairwise comparisons yielded non-significant differences (*p* > 0.05), suggesting that neither historical nor monthly erosion estimates in this transect were substantially influenced by the surveying method (Fig. 7).

These findings indicate that the choice of method significantly influenced erosion estimates only in Transect 1, particularly for historical erosion. Furthermore, across all comparisons (historical and monthly erosion in both transects), the estimates obtained using SfM-AC, SfM-UAS, and TLS were not significantly different from one another, demonstrating consistency among the three HRT methods.

### Methods’ feasibility: quantitative and qualitative insights

The quantitative analysis of the outputs generated by HRT methods offers a clearer understanding of their accuracy and precision. Additional parameters, including orthomosaic resolution, DSM resolution, and point density, were also evaluated. All metrics showed similar performance across the three methods. MAE values were below 1 cm for all methods and both transects, indicating high accuracy in elevation modeling. Consequently, the propagated error in the DoD analyses ranged from 0.9 cm (SfM-UAS) to 1.2 cm (TLS), representing the minimum level of detection. Thus, any topographic change (erosion or deposition) greater than these thresholds was detected and quantified as a net change (Table 2).

**Table 2:** Quantitative parameters used to evaluate the feasibility of the five surveying methods applied in Transects 1 and 2. Equipment costs are expressed in euros (€). *MAE* mean absolute error; *SD* standard deviation; *RMSE* root mean square error. Dashes (-) indicate parameters that could not be obt*ained for the respective method*

Transect	Method	MAE (m)	SD (m)	RMSE (m)	Orthomosaic resolution (mm∙pixel^-1^)	DSM resolution (mm∙pixel^-1^)	DSM point density (points*∙*m^*-2*^)	Time field work (min)	Time processing (min)	Equipment price (€)
1	CSA	-	-	-	-	-	-	80	135	100
	MaxD	-	-	-	-	-	-	50	60	100
	SfM-AC	0.0074	0.0138	0.0138	0.72	1.43	48.8	14	251	446
	SfM-UAS	0.0080	0.0133	0.0136	1.10	1.10	82.4	43	298	847
	TLS	0.0101	0.0144	0.0153	-	10.00	-	120	335	19800
2	CSA	-	-	-	-	-	-	64	108	100
	MaxD	-	-	-	-	-	-	40	48	100
	SfM-AC	0.0078	0.0121	0.0119	0.66	1.33	56.9	9	159	446
	SfM-UAS	0.0058	0.0098	0.0095	0.92	1.83	29.9	32	259	847
	TLS	0.0083	0.0123	0.0128	-	10.00	-	100	268	19800

Fieldwork time also varied across methods. TLS required the most time in the field, followed by CSA and MaxD, while SfM-UAS and SfM-AC involved considerably shorter fieldwork durations. In contrast, data processing time was greatest for TLS, SfM-UAS, and SfM-AC, and shortest for CSA and MaxD. Regarding costs, TLS stood out with a substantially higher equipment price (~€20,000), whereas all other methods had equipment costs below €1000.

Regarding qualitative parameters, the CSA and MaxD methods are clearly easier to implement, as they require minimal human resource training and involve little bureaucratic complexity. In contrast, the HRT methods require technical expertise for both fieldwork and data processing. Additionally, the SfM-UAS method presents further challenges, including bureaucratic requirements for UAS operation (such as flight permits and civil liability insurance) and operational difficulties in trails with dense understory vegetation. These conditions can increase the risk of equipment-related accidents, such as UAS collisions with vegetation.

Another important qualitative factor concerns the influence of vegetation on data acquisition. All the HRT methods may be affected by the presence of low herbaceous vegetation on the trail surface, as the point cloud generated by photogrammetry or TLS may, under certain conditions, capture the elevation of dense herbaceous cover or fallen leaves rather than the actual ground surface. Although filtering techniques, particularly for TLS data, can be applied to remove vegetation, their effectiveness depends on factors such as vegetation density and point cloud resolution. In areas with dense vegetation or fine herbaceous cover (e.g., grasses or leaf litter), filtering may be challenging or incomplete, potentially reducing the accuracy of ground surface reconstruction.

In contrast, this limitation does not apply to the CSA and MaxD methods, as the measuring rod can be positioned beneath the herbaceous layer during data collection. However, these traditional methods are subject to small measurement variations due to slight penetration of the rod into unconsolidated soil or the displacement of loose surface materials such as pebbles, which may introduce millimeter-scale errors (Table 3).

**Table 3:** Comparative overview of the five erosion surveying methods with respect to equipment cost, staffing requirements, bureaucratic demands, operational advantages and limitations, and additional outputs

Criterion	Traditional methods	HRT methods		
	CSA and MaxD	SfM-AC	SfM-UAS	TLS
Human resources	Technicians with basic training	Technicians with minimal training + photogrammetry/GIS specialists	Technicians with training in piloting and certification + photogrammetry/GIS specialists	TLS-trained technicians + specialists in point cloud processing
Bureaucratic requirements	None	None	High: flight permits and civil liability insurance	None
Advantages	Low cost, simplicity, minimal training required	Versatile, low operational risk, no bureaucracy	Broad spatial coverage (in open areas)	Low operational risk, no bureaucracy
Limitations	Low precision, no spatial output	Requires external data processing; affected by herbaceous vegetation	Requires external data processing; obstructed by dense vegetation; UAS accident risk; high bureaucracy; affected by herbaceous vegetation	Very high equipment cost; requires external data processing; affected by herbaceous vegetation
Additional capabilities	Only measures erosion	Generates georeferenced orthomosaics and digital elevation models	Generates georeferenced orthomosaics and digital elevation models	Generates digital elevation models

**Fig. 8:**
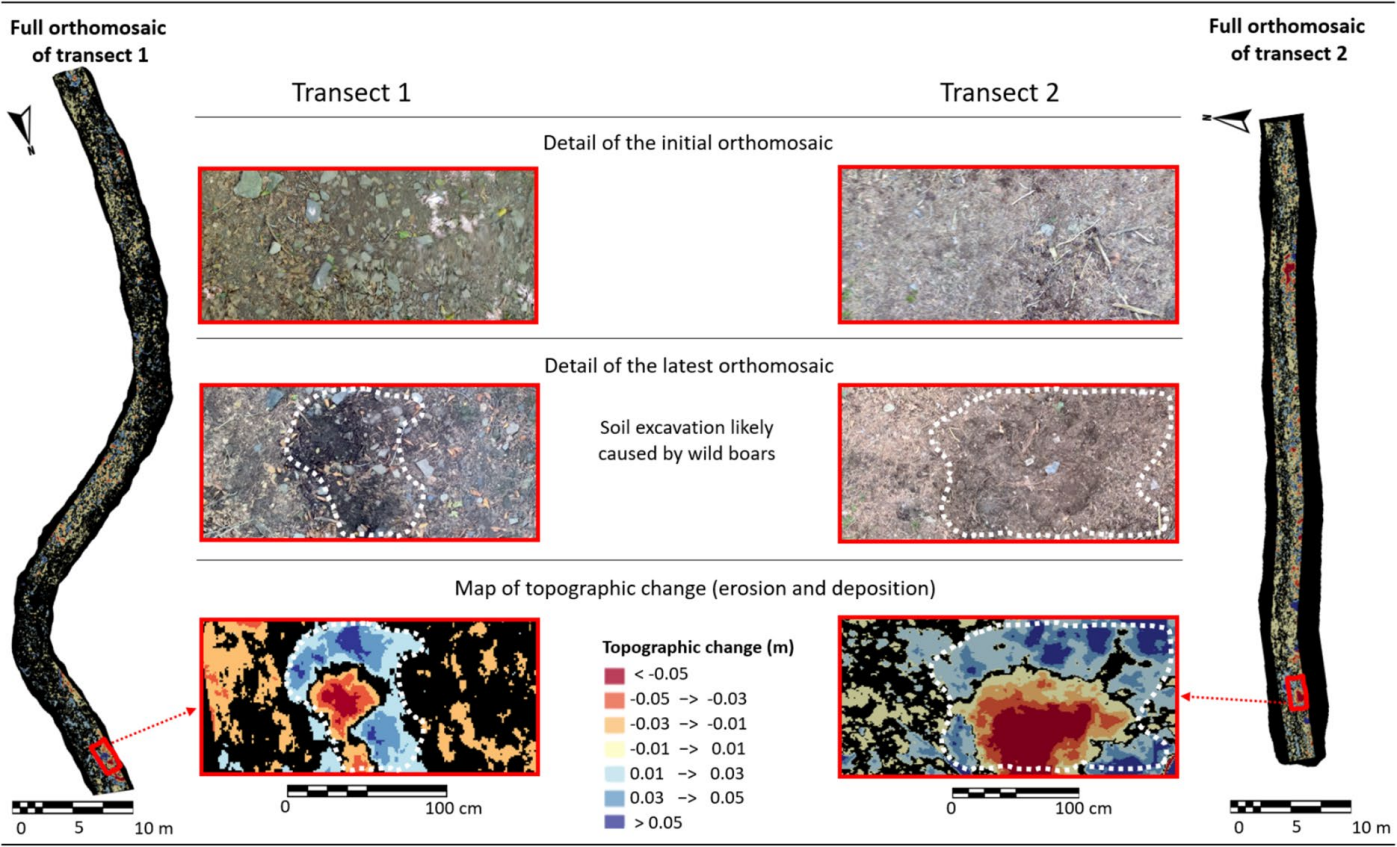
On the left, full orthomosaic of Transect 1. On the right, full orthomosaic of Transect 2. In the center, from top to bottom and within the red rectangles (which correspond to the highlighted areas in the orthomosaics): detail of the initial orthomosaic; detail of the latest orthomosaic (acquired 34 days later); and the corresponding map of topographic change (erosion and deposition), showing a section of Plot 10 in Transect 1 and a section of Plot 2 in Transect 2. In both the latest orthomosaics and the topographic change maps, the area outlined by the white dashed line corresponds to the section affected by soil excavation likely caused by wild boars. At the bottom center, the legend indicates that red, orange, and yellow tones represent the excavated area (erosion), while blue tones indicate deposition zones where the displaced soil was redeposited

Despite requiring substantial technical expertise for both data collection and analysis, the SfM-AC and SfM-UAS methods offer a notable advantage. In addition to generating DSMs and erosion/deposition maps, which are also produced by TLS, SfM methods uniquely provide high-resolution orthomosaics. When overlaid with DSMs, these georeferenced images enhance the identification of fine-scale topographic change patterns along the trail surface. Such patterns, detectable at sub-centimeter resolution, include surface runoff pathways, localized soil damage caused by specific trail uses, and animal excavations, as observed in this study. Together, these spatial patterns offer valuable insights for erosion monitoring, with important implications for both trail management and scientific research (Fig. 8).

## Discussion

Regarding historical and monthly erosion, significant differences among methods were observed only in Transect 1, which is likely associated with its greater surface complexity. This transect exhibited heterogeneous trail tread, including numerous fixed and loose rocks and exposed roots, whereas Transect 2 presented fewer of these elements, resulting in a more uniform surface. Such structural irregularities may disproportionately affect traditional methods (CSA and MaxD) by introducing sampling-related biases. For example, if most CSA or MaxD lines intersect relatively smooth portions of the trail surface, while substantial surface irregularities, such as rocks, exposed roots, or accumulated debris, are present between consecutive cross-sections, erosion may be overestimated. This occurs because such surface irregularities located between consecutive cross-sections are not adequately captured by the sampled profiles, leading to a simplified representation of the actual trail surface. Conversely, if a CSA or MaxD line coincides directly with a large rock or root, the measured surface may be artificially elevated, leading to an underestimation of erosion. These effects arise from the fixed spatial sampling inherent to traditional methods and help explain the higher deviations observed under complex surface conditions. Based on this reasoning, we infer that all methods tend to yield comparable results in trails with low erosion levels and relatively uniform surfaces, such as those lacking pronounced rills, large clasts, or exposed roots. This is typically the scenario found in newer trails or those with limited use.

However, many trails in protected natural areas exhibit high levels of erosion, often characterized by features such as concentrated surface runoff, exposed rocks, and roots (Marion & Leung, 2001). In such cases, erosion estimation using more sensitive methods with greater surface modeling capabilities, such as HRT methods, can provide estimates that are closer to reality (Tomczyk & Ewertowski, 2013). The HRT methods also allow for the calculation of the minimum level of detection (minLoD), as represented by the propagated error in DoD analyses, which can ensure that the detected topographic changes (erosion or deposition) are indeed real and not merely the result of modeling errors inherent to the method.

Regarding monthly erosion, estimates (for both transects) were more consistent across methods, particularly in Transect 2. This pattern underscores the importance of method selection in geomorphological studies, particularly on trails with highly heterogeneous surface conditions and elevated erosion rates, such as those found in Transect 1. In such cases, discrepancies between measurement techniques can introduce substantial variability in erosion estimates. The CSA method, in particular, tended to overestimate erosion relative to the other techniques. This overestimation may stem from its limited spatial resolution and vulnerability to surface irregularities: when cross-sections miss localized deposits or intersect areas of exaggerated surface loss (e.g., loose sediment displacement), the resulting profiles may not accurately reflect actual volumetric changes. In contrast, more uniform trail surfaces, as observed in Transect 2, reduce the impact of such sampling errors, leading to more comparable estimates across methods.

Regarding the methods’ feasibility, based on accuracy (MAE) and precision (SD), the HRT methods demonstrated satisfactory performance in detecting changes in trail surfaces, being capable of identifying variations on the order of 1 cm in both transects. These quantitative metrics, which reflect the methods’ ability to capture net surface change, result from the high-density altimetric data used to generate the DSMs, providing unique elevation values for nearly every square millimeter of the area of interest (see DSM resolution in Table 2).

Although this minimum level of detection is similar in magnitude to vertical errors reported in previous studies, such as Hayakawa et al. (2025), who reported a 1-cm vertical error using a combined photogrammetry and LiDAR approach to assess trail surface deformation, and Salesa et al. (2020), who reported a 1.8-cm vertical error using UAS and smartphone-based photogrammetry to measure trail erosion, these values were based solely on georeferencing errors derived from ground control points (GCPs). Although Tomczyk and Ewertowski (2013) used independent checkpoints to calculate vertical error (RMSE < 1 cm), the present study is the first known in the context of trail management to both quantify vertical uncertainty through independent checkpoint validation and apply this value to calculate propagated error within a DSM of Difference (DoD) framework, thereby enabling a robust and explicit definition of the minimum level of detection.

The calculation of accuracy and precision for the cross-sectional area method is limited by the fact that the method relies on only one measurement every 5 m. Furthermore, the method inherently involves a degree of generalization within each cross-sectional profile, as data are collected at discrete 10-cm intervals rather than continuously. This limitation due to discrete sampling is even more pronounced in the case of the MaxD method. It estimates erosion volume based on a single measurement (maximum depth) and assumes a simplified triangular cross-sectional shape, an approximation that often deviates significantly from the irregular geometry observed in real trail conditions.

In addition, the CSA method is subject to measurement errors, such as slight inconsistencies in depth readings taken every 10 cm, which can arise from small variations in probe placement and from repositioning the horizontal bar between surveys (Tomczyk & Ewertowski, 2013). These sources of observational error alone (i.e., excluding other sources such as extrapolation or methodological assumptions) can result in discrepancies ranging from 2 to 14 mm (Coleman, 1977). To minimize such errors, Coleman (1977) proposed a short-term detailed measurement method that involves installing rigid horizontal bars fixed with concrete along the trail edges. While this approach can increase measurement precision, it also disturbs the original topography and soil matrix due to the use of concrete. In addition, the installation of permanent structures alters the landscape and leaves long-lasting physical markers along the trail. Transporting a long, heavy perforated bar also poses significant logistical difficulties, particularly in wide trail sections, where the bar must be even longer to span the full width. This increases both the weight and the handling complexity of the equipment. Altogether, these limitations make the method costly and impractical for application in remote areas.

Thus, for scientific research purposes, the CSA method presents limitations in determining accuracy, precision, and minimum level of detection. These limitations affect both the estimation of cross-sectional areas and, more importantly, the estimation of total erosion when values are extrapolated along the trail length. However, for practical management of protected natural areas, the method remains useful. When the objective is to identify relevant changes on the order of centimeters over long time scales, the CSA method offers a low-cost and operationally simple approach. It requires limited technical expertise and produces results that are straightforward to interpret, making it feasible for management applications (Coleman, 1977; Salesa et al., 2020).

To date, most scientific studies aiming to quantify erosion on recreational trails in natural areas have relied on the CSA method (e.g., Goeft & Alder, 2001; Marion and Wimpey, 2017; Salesa & Cerdà, 2019; Smith & Pickering, 2025). Some studies have proposed adaptations to reduce field data collection time and enable sampling at more locations. For instance, Olive and Marion (2009) proposed the Variable CSA method, which consists of point sampling using fixed intervals and a random starting point, while measuring only depths greater than 2.5 cm. In this case, the authors acknowledged that the erosion results obtained with this method are underestimations due to the exclusion of erosion with depths less than 2.5 cm.

With a similar objective of evaluating more practical and time-efficient methods for measuring erosion, Salesa and Cerdà (2019) compared estimates derived from the CSA and MaxD methods. They found that MaxD captured approximately 78% of the erosion estimated by the CSA method. This finding aligns with the results of the present study, which also observed that MaxD tends to underestimate erosion compared to CSA. This underestimation occurs because the area of a triangle inscribed within a concavity is inherently smaller than the area of the concavity itself, which represents the true cross-sectional area. Nonetheless, Meadema et al. (2020) advocate using only the maximum incision value, rather than CSA or average incision, to analyze the influence of trail layout on soil loss, emphasizing its lower field time demands and the fact that this variable is not influenced by trail width.

More recent studies, such as Salesa et al. (2020), compared erosion estimates obtained through terrestrial and aerial photogrammetry methods (using smartphone and UAS cameras, respectively) and the CSA method applied at high frequency with measurements taken every meter. Their study was conducted in an area affected by a wildfire that had completely removed the vegetation. The results showed that erosion estimates were very similar across all methods, leading the authors to conclude that CSA was the most applicable technique for measuring trail erosion. This conclusion was based on CSA’s lower cost, minimal training requirements, and its reported applicability even in densely vegetated, forested environments. However, the authors assumed that photogrammetry methods are limited to areas without vegetation, such as arid and semi-arid zones or areas affected by wildfires where vegetation has been completely removed.

Tomczyk et al. (2023) similarly highlighted the operational challenges of using optical remote sensing methods to capture data beneath forest canopy cover. Nonetheless, they noted that ongoing improvements in UAS maneuverability could help address these challenges. In the present study, we demonstrate that both SfM-AC and SfM-UAS approaches are already capable of overcoming this constraint and can be effectively applied in forested environments. The use of UAS with skilled piloting, and especially the action camera mounted on a pole, allows data acquisition beneath the canopy, even if only over a relatively short trail segment (~90 m). Accordingly, and in line with Tomczyk’s perspective, we may expect that continued advances in UAS-based image acquisition will soon enable the practical surveying of longer trail segments under canopy cover, thereby broadening the feasibility of these methods across diverse ecosystems.

Regarding fieldwork time, TLS was the most time-consuming method, as it required scan positions approximately every 10 m along the trail. CSA ranked next in time demand, as it required both preparing the section for measurement and carefully taking measurements every 10 cm along each section. MaxD required only a single depth measurement per cross-section, but still demanded time for setup and positioning, making it moderately time-consuming. In the case of the SfM-UAS method, most of the field time was dedicated to planning the flight route and preparing the equipment, rather than to the short duration of the actual flight for image capture. The most time-efficient method was SfM-AC. This approach required only attaching the camera to a pole, activating a pre-set image capture mode, and walking along the trail margins at a steady pace to ensure consistent image quality.

The inverse pattern in data processing time (longer for HRT methods and shorter for CSA and MaxD) is due to the fact that the former methods require extensive computational analyses, such as generating point clouds and DSMs, in addition to multiple detailed and sequential processing steps across different software platforms. In contrast, the latter methods are processed simply using spreadsheet software capable of performing calculations and data management (e.g., Excel), which is widely used by most researchers and technicians working in protected natural areas (Coleman, 1977; Marion, 2023).

Regarding equipment and software costs, the TLS method stands out, with equipment expenses alone being more than 20 times higher than those of the other methods, representing a significant barrier to its adoption in many contexts and typically being accessible only to research groups with substantial funding, as noted by Salesa and Cerdà (2020). In contrast, equipment costs for the other methods remain under €1000, which is generally not a limiting factor for research institutions or natural area management agencies, as also highlighted by Verma and Bourke (2019) in their study on the use of SfM photogrammetry to generate sub-millimeter-resolution digital elevation models for analyzing rock breakdown features. However, it is important to note that even this level of cost may still represent a barrier in underfunded institutions or in lower-income countries, as also discussed by Stark et al. (2021), who emphasized that access to high-end equipment and the associated operational training remain financially unfeasible for many institutions worldwide. To help offset these limitations, many data processing steps can be conducted using free and open-source software, including image alignment, point cloud generation, and the creation of orthophotos and digital elevation models.

In addition to quantitative parameters, a holistic analysis of method feasibility requires considering qualitative aspects such as the need for skilled human resources, bureaucratic complexity, and the potential to generate results beyond erosion estimation. TLS is the method that demands the highest level of technical expertise, from field data collection to data processing and the generation of final erosion results. Considering its prohibitive cost in many cases, TLS emerges as the least feasible method for measuring and monitoring trail erosion.

In the case of the SfM-UAS method, park managers can receive basic training to operate a UAS and capture trail imagery in the field (Tomczyk et al., 2023). However, the processing and analysis of the data, similarly to the SfM-AC method, may still require external expertise. This reliance on specialized services for data processing, combined with the need to navigate the bureaucratic procedures involved in UAS deployment, reduces the overall feasibility of this approach.

Thus, among the HRT methods, the SfM-AC stands out in terms of feasibility due to its low cost, relatively low demand for personnel, and lack of bureaucratic constraints. Moreover, compared to the CSA and MaxD methods, SfM-AC allows for the quantification of its sensitivity (detection threshold) in detecting topographic change. It also enables the generation of orthomosaics and georeferenced maps of erosion and deposition at millimeter resolution, which support spatial analysis through the visual identification of change patterns along the trail tread.

These findings underscore the importance of selecting appropriate measurement techniques for trail erosion studies. This is particularly relevant in treads with highly heterogeneous surfaces or when spatially explicit, high-resolution mapping is required. In this context, methods like SfM-AC offer significant advantages such as low cost, no bureaucratic constraints, and quantifiable sensitivity. These methods also allow users to generate orthomosaics and georeferenced maps of erosion and deposition, which aid in the visual identification of surface change patterns.

## Limitations and perspectives

The estimation of erosion using the CSA method does not allow for the calculation of accuracy and precision metrics with the tools applied in this study. This limitation stems from the method’s reliance on measurements from cross-sections spaced every 5 m to generalize altimetric variation (or depth) across the entire trail tread area where topographic changes are to be estimated. Consequently, it was not possible to compare these metrics with those obtained from the HRT methods.

Due to the logistical and operational challenges of applying five different erosion measurement methods under similar conditions, this study was limited to a short monitoring period and only to two trails, thereby encompassing a relatively narrow range of environmental conditions and trail characteristics. As a result, the findings may not fully capture the variability in methods’ performance over longer time spans or under broader field conditions. In particular, the scalability of the tested methods to longer trail sections or multi-temporal monitoring campaigns deserves further consideration. Methods such as SfM and TLS differ not only in field and processing time, but also in how they scale to more complex monitoring schemes, including the need for GCPs to ensure spatial consistency across time. These logistical requirements may affect the relative efficiency and practicality of each method depending on the monitoring context.

Future research should therefore extend this comparative approach across longer temporal scales and to trails with more diverse attributes, including variations in slope gradient, slope alignment, substrate type, vegetation cover, lighting conditions (e.g., shadows in forested areas), erosion severity, and the presence of surface obstacles such as rocks and roots. Moreover, for SfM-based methods, the influence of different image acquisition patterns (e.g., parallel, cross-grid, or unstructured) on the quality of derived products should be further investigated. Such efforts would contribute to refining protocols and assessing the broader applicability of each method, ultimately supporting more effective and evidence-based trail monitoring strategies.

## Conclusions

This study demonstrates that erosion estimates are significantly influenced by the surveying method employed. Statistically significant differences were found between the traditional methods (CSA and MaxD), and particularly between these traditional methods and all high-resolution topographic (HRT) methods (SfM-AC, SfM-UAS, and TLS), whereas no significant differences were observed among the HRT methods themselves. Traditional methods (CSA and MaxD) are simple and cost-effective, making them suitable for routine trail management and long-term monitoring. However, as they do not allow for the quantification of measurement uncertainty, they may introduce undetected errors. In contrast, high-resolution topographic (HRT) methods (SfM-AC, SfM-UAS, and TLS) offer greater accuracy and allow for the quantification of measurement uncertainty and detection thresholds. Nonetheless, these methods require technical expertise and, in the case of TLS, entail high equipment costs. Among them, structure from motion with action camera (SfM-AC) stands out as a promising low-cost alternative that balances precision and operational feasibility across diverse environments. Additionally, SfM-AC can generate erosion and deposition maps that support spatially explicit analyses of trail surface change. This makes it particularly valuable for trail monitoring in natural areas, where technical and financial resources are often limited but accurate erosion assessment is essential for informed decision-making. These results emphasize the necessity of adapting the methodology to the study objectives, environmental conditions, and available resources.

## Supplementary Information

Below is the link to the electronic supplementary material.

ESM1 (DOCX 31.2 KB)

## Data Availability

No datasets were generated or analysed during the current study.
