# Correction to: Trail erosion assessment and monitoring in natural areas: a comparison of traditional and high‑resolution topographic surveying methods

**DOI:** 10.1007/s10661-026-15397-9

**Published:** 2026-05-14

**Authors:** Marcos Vinícius Ribeiro de Castro Simão, Estela Inés Farias‑Torbidoni, Víctor Dorado, Manel Llena

**Affiliations:** 1 University of Lleida, Lleida, Spain; 2 National Institute of Physical Education of Catalonia (INEFC), Lleida, Spain; 3 Federal Institute of Education, Science and Technology of Amazonas - Campus Tefe - IFAM, Tefe, Brazil; 4 Social and Educational Research Group On Physical Activity and Sport (GISEAFE), Barcelona, Spain; 5 Fluvial Dynamics Research Group (RIUS), University of Lleida (UdL), Lleida, Spain

**Correction to: Environ Monit Assess (2026) 198:423**

10.1007/s10661-026-15212-5

In the published version of this article, Figure 1 was published incorrectly. The original version submitted included (i) transect overlays represented in pink and (ii) specific image cropping to match the figure caption. In the published version, the transect overlays were removed and the images were improperly cropped. As a result, the figure does not correspond accurately to its caption or to the intended representation of the study design. The corrected version of Figure 1 is provided here.

**Fig. 1:**
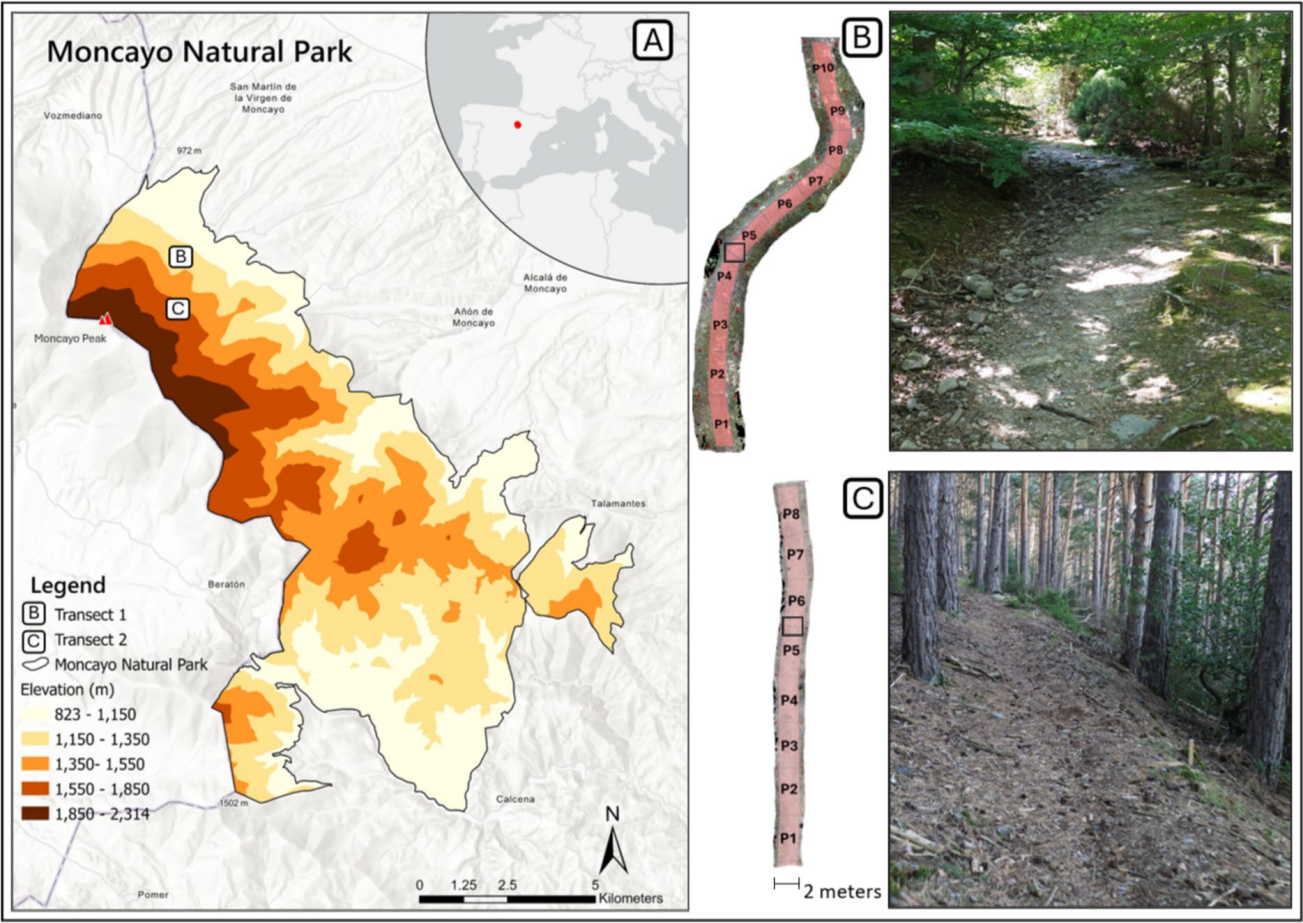
Study area. **A** Location of Moncayo Natural Park, with inset showing its position within Spain and Europe. **B** Sampling plots over the orthomosaic of Transect 1 (left), with black rectangle indicating the area shown in the photo (right), featuring exposed roots and rocks from millimetric fragments to large boulders. **C** Sampling plots over the orthomosaic of Transect 2 (left), with black rectangle indicating the photo area (right), showing fewer exposed elements but abundant pine needles

Additionally, Table 2 was published with an extra column. This alteration disrupted the table’s formatting and readability. The corrected version of Table 2 is provided here.

**Table 2:** Quantitative parameters used to evaluate the feasibility of the five surveying methods applied in Transects 1 and 2. Equipment costs are expressed in euros (€). MAE = mean absolute error; SD = standard deviation; RMSE = root mean square error. Dashes (-) indicate parameters that could not be obtained for the respective method

Transect	Method	MAE (m)	SD (m)	RMSE (m)	Orthomosaic resolution (mm∙pixel^-1^)	DSM resolution (mm∙pixel^-1^)	DSM point density (points*∙*m^*-2*^)	Time field work (min)	Time processing (min)	Equipment price (€)
1	CSA	-	-	-	-	-	-	80	135	100
	MaxD	-	-	-	-	-	-	50	60	100
	SfM-AC	0.0074	0.0138	0.0138	0.72	1.43	48.8	14	251	446
	SfM-UAS	0.0080	0.0133	0.0136	1.10	1.10	82.4	43	298	847
	TLS	0.0101	0.0144	0.0153	-	10.00	-	120	335	19800
2	CSA	-	-	-	-	-	-	64	108	100
	MaxD	-	-	-	-	-	-	40	48	100
	SfM-AC	0.0078	0.0121	0.0119	0.66	1.33	56.9	9	159	446
	SfM-UAS	0.0058	0.0098	0.0095	0.92	1.83	29.9	32	259	847
	TLS	0.0083	0.0123	0.0128	-	10.00	-	100	268	19800

The original article has been corrected.

