# Supplementary material for: Trail erosion assessment and monitoring in natural areas: a comparison of traditional and high-resolution topographic surveying methods
Source: Environ Monit Assess. 2026 Apr 9;198(5):423. doi: 10.1007/s10661-026-15212-5 (PMC13061827; doi:10.1007/s10661-026-15212-5)
Supplement: Supplementary file 1 — (DOCX 31.2 KB) [file 10661_2026_15212_MOESM1_ESM.docx]

**SUPPLEMENTARY MATERIAL**

**Table 1**

Relevant information about the devices used to capture images ([DJI Mini 3](https://www.dji.com/es/mini-3/specs) and [GoPro Hero 12 Black](https://gopro.com/en/us/shop/cameras/hero12-black/CHDHX-121-master.html?srsltid=AfmBOooY8JS3OLBx5iXWp8UzugSPun4bTeP5h0KEPJYh6D1PqXua70zq)) for the structure-from-motion process, based on data from the official websites.

| Feature | DJI Mini 3 Pro | GoPro Hero 12 Black |
| --- | --- | --- |
| Model | DJI Mini 3 Pro | GoPro Hero 12 Black |
| Weight | 248 g | 153 g |
| Dimensions (Folded/Standard) | 148 × 90 × 62 mm | 71 × 55 × 34 mm |
| Max Speed (Horizontal) | 16 m∙s^-1^ | N/A |
| Max Flight/Operational Time | 38 minutes (flight time) | Up to 155 minutes of continuous recording at 1080p30 |
| Max Flight/Operational Distance | 18 km | N/A |
| Wind Resistance | Up to 10.7 m∙s^-1^ | N/A |
| Operating Temperature | -10 to 40 °C | -10 to 35 °C |
| GNSS (GPS System) | GPS + GLONASS + Galileo | N/A |
| Image Sensor | 1/1.3-in CMOS, Effective pixels: 48 MP (8064 × 6048 image size) | 1/1.9" CMOS, 27.6 MP active pixels (5599x4927 image size) |
| Lens | 24 mm equivalent focal length, FOV 82.1°. Focus from 1 m to ∞ | 35 mm equivalent focal length, FOV 156° |
| f-value | f/1.7 | f/2.5 |
| Focal length | 6.72 mm | 2.71 mm |
| Photo intervals (seconds) | 2 / 3 / 5 / 7 / 10 / 15 / 20 / 30 / 60 | 0.5 / 1 / 2 / 3 / 10 / 30 / 60 / 120 |
| Stabilization | 3-axis mechanical (tilt, roll, pan) | HyperSmooth 6.0 stabilization |
| Hovering Accuracy (if applicable) | ±0.1 m with visual, ±0.5 m with GNSS (vertical); ±0.3 m with visual, ±1.5 m with GNSS (horizontal) | N/A |
| Additional Features | Vision system underneath drone for detection | Waterproof, supports live streaming, advanced power management |

**Table 2**

General data for each transect and corresponding historical and monthly erosion values obtained using each method (CSA, MaxD, SfM-AC, SfM-UAS, TLS).

| Transect | Plot | Plot area (m2) | Plot length (m) | CSA (m^3^.m^-2^) | | MaxD (m^3^.m^-2^) | | SfM-AC (m^3^.m^-2^) | | SfM-UAS (m^3^.m^-2^) | | TLS (m^3^.m^-2^) | |
| --- | --- | --- | --- | --- | --- | --- | --- | --- | --- | --- | --- | --- | --- |
|  |  |  |  | historical | monthly | historical | monthly | historical | monthly | historical | monthly | historical | monthly |
| T1 | 1 | 11.64 | 5.82 | -0.30293 | -0.02327 | -0.19284 | -0.00618 | -0.09190 | -0.00215 | -0.10051 | -0.00165 | -0.10622 | -0.00545 |
|  | 2 | 8.91 | 4.46 | -0.20004 | -0.01555 | -0.13436 | -0.00446 | -0.01648 | -0.00284 | -0.01508 | -0.00181 | -0.01951 | -0.00418 |
|  | 3 | 8.73 | 4.36 | -0.15186 | -0.00143 | -0.10059 | -0.00150 | -0.04333 | -0.00032 | -0.04361 | 0.00064 | -0.04843 | -0.00089 |
|  | 4 | 8.82 | 4.41 | -0.20791 | -0.01305 | -0.14427 | -0.00368 | -0.06436 | -0.00577 | -0.06740 | -0.00293 | -0.07120 | -0.00529 |
|  | 5 | 9.96 | 4.98 | -0.20084 | -0.03564 | -0.16447 | -0.00507 | -0.01236 | -0.00513 | -0.01006 | -0.00597 | -0.01761 | -0.01312 |
|  | 6 | 11.00 | 5.50 | -0.09728 | -0.03399 | -0.09196 | -0.00832 | 0.00156 | -0.00360 | 0.00866 | -0.00302 | 0.00270 | -0.00748 |
|  | 7 | 11.15 | 5.58 | -0.03968 | -0.00325 | -0.03455 | 0.01520 | 0.00543 | -0.00216 | 0.01419 | -0.00057 | 0.00871 | -0.00373 |
|  | 8 | 11.26 | 5.63 | -0.10669 | 0.00164 | -0.07645 | 0.02037 | -0.00590 | -0.00321 | -0.00036 | -0.00166 | -0.00347 | -0.00322 |
|  | 9 | 9.95 | 4.98 | -0.14462 | -0.01797 | -0.12056 | -0.00438 | -0.04414 | -0.00544 | -0.04319 | -0.00375 | -0.04716 | -0.00624 |
|  | 10 | 11.78 | 5.89 | -0.20193 | -0.01201 | -0.15376 | -0.01677 | -0.07955 | -0.00746 | -0.08494 | -0.00162 | -0.09901 | -0.01624 |
| Mean T1 |  |  |  | -0.16538 | -0.01545 | -0.12138 | -0.00148 | -0.03510 | -0.00381 | -0.03423 | -0.00223 | -0.04012 | -0.00658 |
| T2 | 1 | 10.20 | 5.10 | -0.10766 | -0.03804 | -0.08438 | -0.02289 | -0.06569 | -0.00801 | -0.05156 | 0.00602 | -0.06249 | -0.00450 |
|  | 2 | 9.39 | 4.70 | -0.05499 | -0.00980 | -0.05921 | 0.01277 | -0.05330 | 0.00712 | -0.05511 | 0.00647 | -0.05114 | 0.01000 |
|  | 3 | 9.89 | 4.94 | -0.05943 | -0.00624 | -0.05404 | 0.01903 | -0.16240 | -0.00260 | -0.15616 | 0.00474 | -0.16264 | -0.00229 |
|  | 4 | 10.37 | 5.19 | -0.08106 | -0.00168 | -0.06378 | -0.00190 | -0.03152 | -0.00449 | -0.03260 | -0.00568 | -0.03585 | -0.00761 |
|  | 5 | 9.76 | 4.88 | -0.13053 | 0.00753 | -0.08925 | 0.00392 | -0.16945 | -0.00339 | -0.16897 | -0.00307 | -0.17213 | -0.00573 |
|  | 6 | 9.62 | 4.81 | -0.12111 | 0.03307 | -0.08691 | 0.02064 | -0.26927 | -0.00884 | -0.26350 | -0.00366 | -0.26928 | -0.00887 |
|  | 7 | 9.95 | 4.97 | -0.08560 | 0.02622 | -0.08033 | 0.01555 | -0.08871 | -0.01073 | -0.08225 | -0.00514 | -0.08348 | -0.00570 |
|  | 8 | 9.69 | 4.85 | -0.07776 | -0.00810 | -0.07689 | 0.00039 | -0.21383 | -0.02139 | -0.20734 | -0.01564 | -0.18567 | 0.00646 |
| Mean T2 |  |  |  | -0.08977 | 0.00037 | -0.07435 | 0.005939 | -0.13177 | -0.00654 | -0.12719 | -0.00199 | -0.12783 | -0.00228 |

**Table 3**

Propagated error associated with each pair of DSMs used in the DoD analysis. Each row represents a specific DSM pair comparison for one of the HRT methods and the corresponding propagated error. ROS refers to the reconstructed original surface, based on the initial DSM-UAS reconstructed using the Topo to Raster interpolation method. T1 and T2 refer to Transects 1 and 2, respectively.

| Transect | Erosion period | DSM pair for DoD Analysis | Propagated error (m) |
| --- | --- | --- | --- |
| 1 | Historical erosion | DSM_ROS_T1 -> DSM_Latest_T1_SfM-AC | 0.01085 |
|  |  | DSM_ROS_T1 -> DSM_Latest_T1_SfM-UAS | 0.01124 |
|  |  | DSM_ROS_T1 -> DSM_Latest_T1_TLS | 0.01287 |
|  | Monthly erosion | DSM_Initial_T1 -> DSM_Latest_T1_SfM-AC | 0.01085 |
|  |  | DSM_Initial_T1 -> DSM_Latest_T1_SfM-UAS | 0.01124 |
|  |  | DSM_Initial_T1 -> DSM_Latest_T1_SfM-TLS | 0.01287 |
| 2 | Historical erosion | DSM_ROS_T2 -> DSM_Latest_T2_SfM-AC | 0.00971 |
|  |  | DSM_ROS_T2 -> DSM_Latest_T2_SfM-UAS | 0.00820 |
|  |  | DSM_ROS_T2 -> DSM_Latest_T2_TLS | 0.01015 |
|  | Monthly erosion | DSM_Initial_T2 -> DSM_Latest_T2_SfM-AC | 0.00971 |
|  |  | DSM_Initial_T2 -> DSM_Latest_T2_SfM-UAS | 0.00820 |
|  |  | DSM_Initial_T2 -> DSM_Latest_T2_SfM-TLS | 0.01015 |

**Table 4**

Results (p-values) of the Shapiro-Wilk normality test, Levene’s test for homogeneity of variance, and Type III ANOVA with Satterthwaite's method for historical and monthly erosion in Transects 1 and 2.

| Dataset | Shapiro-Wilk normality test  (p-value) | Levene's Test for Homogeneity of Variance (p-value) | Type III Analysis of Variance Table with Satterthwaite's method (p-value) |
| --- | --- | --- | --- |
| Transect 1 - Historical erosion | 0.1281 | 0.1915 | 4.96e-16 *** |
| Transect 1 - Monthly erosion | 0.02674* | 0.004903 ** | 0.0001528 *** |
| Transect 2 - Historical erosion | 0.01587* | 4.636e-05*** | 0.03567 * |
| Transect 2 - Monthly erosion | 0.1178 | 0.06971 | 0.3945 |

**Table 5**

Results (p-values) of Tukey’s HSD and pairwise t-tests without assuming equal variances, comparing historical and monthly erosion across all method pairs for Transects 1 and 2.

| Dataset | Contrast | Tukey’s HSD test (p-value) | pairwise t-tests without assuming equal variances |
| --- | --- | --- | --- |
| Transect 1 - Historical erosion | CSA - MaxD | 0.0017 * | - |
|  | CSA - SfM-AC | <.0001 * | - |
|  | CSA - SfM-UAS | <.0001 * | - |
|  | CSA - TLS | <.0001 * | - |
|  | MaxD - SfM-AC | <.0001 * | - |
|  | MaxD - SfM-UAS | <.0001 * | - |
|  | MaxD - TLS | <.0001 * | - |
|  | SfM-AC - SfM-UAS | 1.0000 | - |
|  | SfM-AC - TLS | 0.9892 | - |
|  | SfM-UAS - TLS | 0.9804 | - |
| Transect 1 - Monthly erosion | CSA – MaxD | 0.0003 * | 0.0177 * |
|  | CSA - SfM-AC | 0.0027 * | 0.0185 * |
|  | CSA - SfM-UAS | 0.0006 * | 0.0097 * |
|  | CSA - TLS | 0.0333 * | 0.0633 . |
|  | MaxD - SfM-AC | 0.9294 | 0.5273 |
|  | MaxD - SfM-UAS | 0.999 | 0.8355 |
|  | MaxD - TLS | 0.4172 | 0.2023 |
|  | SfM-AC - SfM-UAS | 0.9825 | 0.0919 . |
|  | SfM-AC - TLS | 0.8742 | 0.1122 |
|  | SfM-UAS - TLS | 0.5741 | 0.0184 * |
| Transect 2 - Historical erosion | CSA – MaxD | 0.9527 | 0.186 |
|  | CSA - SfM-AC | 0.2895 | 0.218 |
|  | CSA - SfM-UAS | 0.4096 | 0.264 |
|  | CSA - TLS | 0.3914 | 0.246 |
|  | MaxD - SfM-AC | 0.0585 . | 0.099 . |
|  | MaxD - SfM-UAS | 0.1008 | 0.121 |
|  | MaxD - TLS | 0.0937 . | 0.109 |
|  | SfM-AC - SfM-UAS | 0.9995 | 0.915 |
|  | SfM-AC - TLS | 0.9998 | 0.926 |
|  | SfM-UAS - TLS | 1 | 0.988 |
| Transect 2 - Monthly erosion | CSA – MaxD | 0.8988 | - |
|  | CSA - SfM-AC | 0.8037 | - |
|  | CSA - SfM-UAS | 0.9954 | - |
|  | CSA - TLS | 0.9929 | - |
|  | MaxD - SfM-AC | 0.2963 | - |
|  | MaxD - SfM-UAS | 0.7136 | - |
|  | MaxD - TLS | 0.6866 | - |
|  | SfM-AC - SfM-UAS | 0.9488 | - |
|  | SfM-AC - TLS | 0.9592 | - |
|  | SfM-UAS - TLS | 1 | - |
